# Intravenous Thrombolysis Before Endovascular Treatment in Posterior Circulation Occlusions: A MR CLEAN Registry Study

**DOI:** 10.1161/STROKEAHA.123.043777

**Published:** 2024-01-04

**Authors:** Robrecht R.M.M. Knapen, F. Anne V. Pirson, Lucianne C.M. Langezaal, Josje Brouwer, Charles B.L.M. Majoie, Bart J. Emmer, Jan-Albert Vos, Pieter-Jan van Doormaal, Albert J. Yoo, Agnetha A.E. Bruggeman, Geert J. Lycklama à Nijeholt, Chirstiaan van der Leij, Robert J. van Oostenbrugge, Wim H. van Zwam, Wouter J. Schonewille

**Affiliations:** Department of Radiology and Nuclear Medicine (R.R.M.M.K., C.v.d.L., W.H.v.Z.), Maastricht University Medical Center, the Netherlands.; Department of Neurology (R.J.v.O.), Maastricht University Medical Center, the Netherlands.; School for Cardiovascular Diseases Maastricht (CARIM), Maastricht University, the Netherlands (R.R.M.M.K., R.J.v.O., W.H.v.Z.).; Department of Neurology (F.A.V.P.), Haaglanden MC, Hague, the Netherlands.; Department of Radiology (G.J.L.à.N.), Haaglanden MC, Hague, the Netherlands.; Department of Radiology (L.C.M.L., J.-A.V.), St. Antonius Hospital, Nieuwegein, the Netherlands.; Department of Neurology (W.J.S.), St. Antonius Hospital, Nieuwegein, the Netherlands.; Department of Neurology (J.B.), Amsterdam University Medical Center, University of Amsterdam, the Netherlands.; Department of Radiology and Nuclear Medicine (B.J.E.), Amsterdam University Medical Center, University of Amsterdam, the Netherlands.; Department of Radiology and Nuclear Medicine, Amsterdam University Medical Center, University of Amsterdam, the Netherlands (C.B.L.M.M., A.A.E.B.).; Department of Radiology, Erasmus Medical Center, University Medical Center, Rotterdam, the Netherlands (P.-J.v.D.).; Department of Radiology/Neurointervention, Texas Stroke Institute, Dallas-Fort Worth (A.J.Y).; (Department of Neurology, Erasmus MC University; (Department of Radiology, Erasmus MC University Medical Center; (Department of Neurology, Amsterdam UMC, University of Amsterdam, Amsterdam; Department of Neurology, Haaglanden MC, the Hague; Department of Radiology and Nuclear Medicine, Amsterdam UMC, University of Amsterdam; Departments of Neurology and Radiology, Erasmus MC University Medical Center.; Department of Neurology, and Department of Radiology, Maastricht University Medical Center and Cardiovascular Research Institute Maastricht (CARIM); Department of Radiology, Erasmus MC University Medical Center; Department of Radiology and Nuclear Medicine, Amsterdam UMC, University of Amsterdam; Departments of Neurology, Radiology, and Public Health, Erasmus MC University Medical Center; Departments of Neurology and Radiology Maastricht University Medical Center and Cardiovascular Research Institute Maastricht (CARIM).; Department of Neurology, Erasmus MC University Medical Center; Department of Neurology, Erasmus MC University Medical Center; Department of Radiology Erasmus MC University Medical Center; Department of Neurology Amsterdam UMC, University of Amsterdam, Amsterdam; Department of Neurology Leiden University Medical Center; Department of Radiology Leiden University Medical Center; Department of Radiology Leiden University Medical Center; Department of Neurology, Maastricht University Medical Center and Cardiovascular Research Institute Maastricht [CARIM]; Department of Neurology, Rijnstate Hospital, Arnhem; Department of Radiology, Rijnstate Hospital, Arnhem; Department of Neurology Haaglanden MC, the Hague; Department of Neurology HAGA Hospital, the Hague; Department of Radiology, HAGA Hospital, the Hague; Department of Neurology, University Medical Center Utrecht; Department of Radiology University Medical Center Utrecht; Department of Neurology Radboud University Medical Center, Nijmegen; Department of Neurosurgery Radboud University Medical Center, Nijmegen; Department of Neurology, Isala Klinieken, Zwolle; Department of Neurology Elisabeth-TweeSteden ziekenhuis, Tilburg; Department of Neurology Elisabeth-TweeSteden ziekenhuis, Tilburg; Department of Radiology, Elisabeth-TweeSteden ziekenhuis, Tilburg; Department of Neurology, Isala Klinieken, Zwolle; Department of Neurology Isala Klinieken, Zwolle; Department of Radiology Isala Klinieken, Zwolle; Department of Neurology Reinier de Graaf Gasthuis, Delft; Department of Radiology Reinier de Graaf Gasthuis, Delft; Department of Neurology University Medical Center Groningen; Department of Radiology University Medical Center Groningen; Department of Radiology University Medical Center Groningen; Department of Neurology Atrium Medical Center, Heerlen; Department of Radiology Atrium Medical Center, Heerlen; Department of Neurology, Catharina Hospital, Eindhoven; Department of Radiology, Catharina Hospital, Eindhoven; Department of Neurology Isala Klinieken, Zwolle; Department of Neurology, Medisch Spectrum Twente, Enschede; Department of Neurology, Medisch Spectrum Twente, Enschede; Department of Radiology, Leiden University Medical Center; Department of Radiology and Nuclear Medicine; Department of Radiology and Nuclear Medicine, Amsterdam UMC, University of Amsterdam; Department of Radiology and Nuclear Medicine, Amsterdam UMC, University of Amsterdam; Department of Radiology and Nuclear Medicine Amsterdam UMC, University of Amsterdam; Department of Radiology, Maastricht University Medical Center and Cardiovascular Research Institute Maastricht [CARIM]),; Department of Radiology and Nuclear Medicine Amsterdam UMC, University of Amsterdam; Department of Radiology Haaglanden MC, the Hague; Department of Radiology, Haaglanden MC, the Hague; Department of Radiology, Leiden University Medical Center); Department of Radiology, Rijnstate Hospital, Arnhem; Department of Radiology, Catharina Hospital, Eindhoven; Department of Radiology, Amsterdam UMC, Vrije Universiteit van Amsterdam, Amsterdam; Department of Radiology Radboud University Medical Center, Nijmegen; Department of Radiology Haaglanden MC, the Hague; Department of Radiology University Medical Center Groningen; Department of Radiology, Noordwest Ziekenhuisgroep, Alkmaar; Department of Radiology, Catharina Hospital, Eindhoven; Department of Radiology, Elisabeth-TweeSteden ziekenhuis, Tilburg; Department of Neurosurgery Radboud University Medical Center, Nijmegen; Department of Radiology, University Medical Center Utrecht; Department of Radiology Albert Schweitzer Hospital, Dordrecht; Department of Radiology, University Medical Center Groningen; Department of Radiology, HAGA Hospital, the Hague; Department of Neurology Radiology, Radboud University Medical Center, Nijmegen; Department of Radiology, University Medical Center Groningen; Department of Neurosurgery, Radboud University Medical Center, Nijmegen; Department of Radiology, Erasmus MC University Medical Center; Department of Radiology, Maastricht University Medical Center and Cardiovascular Research Institute Maastricht [CARIM]; Department of Neurology, Erasmus MC University Medical Center; Department of Radiology Erasmus MC University Medical Center; Department of Neurology, Amsterdam UMC, University of Amsterdam; Department of Neurology, Haaglanden MC, the Hague; Department of Neurology, Rijnstate Hospital, Arnhem; Department of Radiology, Rijnstate Hospital, Arnhem; Department of Neurology, University Medical Center Utrecht; Department of Radiology, University Medical Center Utrecht; Department of Radiology, Rijnstate Hospital, Arnhem; Department of Radiology Isala Klinieken, Zwolle; Department of Public Health Erasmus MC University Medical Center; Department of Neurology, Erasmus MC University Medical Center; Department of Neurology, Erasmus MC University Medical Center; Department of Neurology, St. Antonius Hospital, Nieuwegein; Department of Neurology Amsterdam UMC, University of Amsterdam; Department of Neurology Rijnstate Hospital, Arnhem; Department of Neurology Rijnstate Hospital, Arnhem; Department of Radiology Rijnstate Hospital, Arnhem; Department of Neurology Haaglanden MC, the Hague; Department of Neurology Haaglanden MC, the Hague; Department of Neurology Radboud University Medical Center, Nijmegen; Department of Neurology Isala Klinieken, Zwolle; Department of Neurology Atrium Medical Center, Heerlen; Department of Neurology University Medical Center Groningen; Department of Neurology University Medical Center Groningen; Department of Neurology,Catharina Hospital, Eindhoven; Department of Neurology Reinier de Graaf Gasthuis; Department of Neurology Medisch Spectrum Twente, Enschede; Department of Neurology Medisch Spectrum Twente, Enschede; Department of Neurology Medisch Spectrum Twente, Enschede; Department of Neurology Maastricht University Medical Center and Cardiovascular Research Institute Maastricht [CARIM]; Department of Neurology Maastricht University Medical Center and Cardiovascular Research Institute Maastricht [CARIM]; Department of Neurology Maastricht University Medical Center and Cardiovascular Research Institute Maastricht [CARIM]; Department of Neurology Leiden University Medical Center; Department of Neurology HAGA Hospital, the Hague; Department of Neurology HAGA Hospital, the Hague; Department of Neurology Rijnstate Hospital, Arnhem; Department of Neurology University Medical Center Utrecht; Department of Neurology Elisabeth-TweeSteden ziekenhuis, Tilburg; Department of Neurology Isala Klinieken, Zwolle; Department of Neurology Radboud University Medical Center, Nijmegen; Department of Neurology Radboud University Medical Center, Nijmegen; Department of Radiology Leiden University Medical Center; Department of Public Health; Departments of Neurology and Public Health Erasmus MC University Medical Center; Department of Radiology and Nuclear Medicine, Amsterdam UMC, University of Amsterdam; Department of Neurology Radboud University Medical Center, Nijmegen; Department of Neurology, Erasmus MC University Medical Center; Department of Neurology Erasmus MC University Medical Center; Department of Neurology, Erasmus MC University Medical Center; Department of Neurology Erasmus MC University Medical Center; Department of Neurology, Erasmus MC University Medical Center; Department of Radiology and Nuclear Medicine, Amsterdam UMC, University of Amsterdam; Department of Radiology, Maastricht University Medical Center and Cardiovascular Research Institute Maastricht [CARIM]; Department of Radiology and Nuclear Medicine Amsterdam UMC, University of Amsterdam; Department of Radiology and Nuclear Medicine Amsterdam UMC, University of Amsterdam; Department of Radiology and Nuclear Medicine; Department of Radiology and Nuclear Medicine Amsterdam UMC, University of Amsterdam; Department of Radiology and Nuclear Medicine Amsterdam UMC, University of Amsterdam; Department of Radiology and Nuclear Medicine Amsterdam UMC, University of Amsterdam; Department of Radiology and Nuclear Medicine, Biomedical Engineering & Physics, Amsterdam UMC, University of Amsterdam; Department of Radiology and Nuclear Medicine Amsterdam UMC, University of Amsterdam; Department of Radiology and Nuclear Medicine Amsterdam UMC, University of Amsterdam; Department of Radiology and Nuclear Medicine Amsterdam UMC, University of Amsterdam; Department of Neurology Rijnstate Hospital, Arnhem; Department of Radiology and Nuclear Medicine Amsterdam UMC, University of Amsterdam; Departments of Neurology and Radiology University Medical Center Groningen; Department of Neurology Amsterdam UMC, University of Amsterdam, Amsterdam; Department of Neurology Amsterdam UMC, University of Amsterdam, Amsterdam; Department of Biomedical Engineering & Physics; Department of Biomedical Engineering & Physics; Department of Biomedical Engineering & Physics Amsterdam UMC, University of Amsterdam; Department of Biomedical Engineering & Physics Amsterdam UMC, University of Amsterdam),; Departments of Neurology Amsterdam UMC, University of Amsterdam

**Keywords:** posterior circulation, stroke, thrombectomy, thrombolytic therapy

## Abstract

**BACKGROUND:**

The effectiveness of intravenous thrombolysis (IVT) before endovascular treatment (EVT) has been investigated in randomized trials and meta-analyses. These studies mainly concerned anterior circulation occlusions. We aimed to investigate clinical, technical, and safety outcomes of IVT before EVT in posterior circulation occlusions in a nationwide registry.

**METHODS:**

Patients were included from the MR CLEAN Registry (Multicenter Randomized Clinical Trial of Endovascular Treatment for Acute Ischemic Stroke in the Netherlands), a nationwide, prospective, multicenter registry of patients with acute ischemic stroke due to a large intracranial vessel occlusion receiving EVT between 2014 and 2019. All patients with a posterior circulation occlusion were included. Primary outcome was a shift toward better functional outcome on the modified Rankin Scale at 90 days. Secondary outcomes were favorable functional outcome (modified Rankin Scale scores, 0–3), occurrence of symptomatic intracranial hemorrhages, successful reperfusion (extended Thrombolysis in Cerebral Ischemia ≥2B), first-attempt successful reperfusion, and mortality at 90 days. Regression analyses with adjustments based on univariable analyses and literature were applied.

**RESULTS:**

A total of 248 patients were included, who received either IVT (n=125) or no IVT (n=123) before EVT. Results show no differences in a shift on the modified Rankin Scale (adjusted common odds ratio, 1.04 [95% CI, 0.61–1.76]). Although symptomatic intracranial hemorrhages occurred more often in the IVT group (4.8% versus 2.4%), regression analysis did not show a significant difference (adjusted odds ratio, 1.65 [95% CI, 0.33–8.35]). Successful reperfusion, favorable functional outcome, first-attempt successful reperfusion, and mortality did not differ between patients treated with and without IVT.

**CONCLUSIONS:**

We found no significant differences in clinical, technical, and safety outcomes between patients with a large vessel occlusion in the posterior circulation treated with or without IVT before EVT. Our results are in line with the literature on the anterior circulation.

Intravenous thrombolysis (IVT) before endovascular treatment (EVT) is recommended in all patients with ischemic stroke due to an intracranial large vessel occlusion (LVO) in the anterior circulation within 4.5 hours after symptom onset.^1^ Although treatment with IVT between 4.5 and 9 hours may be considered in the presence of a mismatch on computed tomography (CT) perfusion in the anterior circulation, there is no consensus about the indication for IVT before EVT in this late time window.^1^

Recent meta-analyses and randomized clinical trials found no superiority or noninferiority in functional outcome and mortality at 90 days between patients with an LVO treated with and without IVT before EVT.^2–8^ These studies mainly concerned patients with anterior circulation occlusions.

The BEST (Basilar Artery Occlusion Endovascular Intervention Versus Standard Medical Treatment), BASICS (Basilar Artery International Cooperation Study), ATTENTION (Endovascular Treatment for Acute Basilar Artery Occlusion: A Multicentre Randomised Clinical Trial), and BAOCHE (Basilar Artery Occlusion Chinese Endovascular) trials are randomized clinical trials on the effectiveness of EVT in patients with basilar artery occlusion (BAO).^9–12^ ATTENTION and BAOCHE showed a beneficial effect of EVT in patients treated within 12 hours and between 6 and 24 hours of symptom onset, respectively. However, no randomized clinical trials are available on the effectiveness of IVT in posterior circulation occlusions.^13^ Two meta-analyses, based on cohort studies, showed lower incidences of intracranial hemorrhage in patients treated with IVT alone for posterior circulation stroke as compared with anterior circulation stroke. These meta-analyses included all posterior circulation occlusions (intracranial vertebral, basilar, and posterior cerebral artery occlusions).^14,15^ In patients with posterior circulation stroke compared with anterior circulation stroke treated with IVT, but without EVT, higher mortality rates were found.^15^ When patients were treated with IVT before EVT, symptomatic intracranial hemorrhage (sICH) rates were comparable and mortality rates were higher in the posterior circulation occlusion as compared with anterior circulation occlusion.^15^

Because the available data from the literature is limited, our study aimed to investigate the outcomes of patients with posterior circulation occlusion treated with EVT, with or without prior IV r-tPA (intravenous recombinant tissue-type plasminogen activator) in a large nationwide registry MR CLEAN Registry (Multicenter Randomized Clinical Trial of Endovascular Treatment for Acute Ischemic Stroke in the Netherlands).^16^

## METHODS

The corresponding author had full access to all the data in this study and takes responsibility for its integrity and the data analysis. Source data will not be made available because of legislative issues on patient privacy. Detailed statistical analyses and analytic methods will be made available on reasonable request to the corresponding author. This study was conducted using the STROBE (Strengthening the Reporting of Observational Studies in Epidemiology) guidelines.

### Design and Participants

Patients were included from the MR CLEAN Registry: a prospective, observational study in all EVT performing centers (n=18) in the Netherlands. The registry included patients treated with EVT for acute ischemic stroke due to LVO between March 2014 and December 2018. The MR CLEAN Registry study protocol was evaluated by the medical ethics committee of the Erasmus University Medical Center and permission was granted to carry out the study as a registry. The need for obtaining informed consent was waived. For the current study, the following inclusion criteria were used: age ≥18 years, National Institutes of Health Stroke Scale score ≥2, and an occlusion in the posterior circulation (intracranial vertebral artery, basilar artery, or posterior cerebral artery) confirmed by CT angiography. Patients in whom only a catheterization was performed due to no intracranial access of the materials were excluded.

The time of symptom onset was defined as the first moment of start symptoms when witnessed or the time the patient was last seen well if the onset was unwitnessed. In patients with mild neurological symptoms with secondary clinical worsening, the time of deterioration was considered as the estimated time of the LVO.

### Treatment

Because EVT was not yet been proven to be effective in BAO patients, EVT was not performed routinely and was not supported by national guidelines. Its use was based on the clinical judgment of the treating physician. All patients with BAO treated with EVT in the Netherlands outside the BASICS trial were registered. IV r-tPA (alteplase) could be administered within 4.5 hours after the estimated time of the LVO but also after 4.5 hours. Treating physicians were free to choose which materials and thrombectomy techniques they used during EVT.

### Outcome Measures

The primary outcome was the modified Rankin Scale (mRS) score at 90 days follow-up, ranging from 0 (no disability) to 6 (death). In the MR CLEAN Registry, the mRS score at 90 days was scored by trained nurses during an in-person or telephonic interview. Secondary outcomes were favorable functional outcome (defined as mRS scores, 0–3), functional independent outcome (defined as mRS scores, 0–2), and the National Institutes of Health Stroke Scale score at 24 to 48 hours. Technical outcomes included procedure duration (defined as groin puncture to reperfusion), first-attempt successful reperfusion, and successful reperfusion. Safety outcomes were the occurrence of sICH within 3 days after EVT, mortality at 90 days, and serious adverse events (eg, stroke progression and pneumonia).

### Imaging Assessment

Intracranial hemorrhage was defined as symptomatic when the patient had neurological deterioration (at least 4 points increase on the National Institutes of Health Stroke Scale) in combination with a hemorrhage (according to the Heidelberg criteria), which was related to the clinical deterioration. An adverse event committee evaluated the medical reports and imaging to determine a sICH.

Reperfusion status was scored on digital subtraction angiography according to the extended Thrombolysis in Cerebral Ischemia (eTICI) by an independent core laboratory. This core laboratory consisted of 8 interventional radiologists or neuroradiologists, all blinded to the clinical findings. The neuroimaging scoring was centrally conducted and the core laboratory had access to all available neuroimaging per patient, this included digital subtraction angiography, noncontrast CT, CT angiography, and CT perfusion when available. The eTICI ranges from 0 (no reperfusion) to 3 (complete reperfusion). In this study, successful reperfusion was defined as eTICI ≥2B (50%–90% reperfusion of affected area), excellent reperfusion as eTICI 3, and first-attempt successful recanalization as eTICI ≥2C (90%–99% reperfusion of affected area) in combination with 1 attempt. When only a digital subtraction angiography was performed because of recanalization, it was registered as early recanalization. When the digital subtraction angiography was made in only 1 direction, the maximum eTICI score was set at 2A. The posterior circulation Acute Stroke Prognosis Early Computed Tomography Score was scored on noncontrast CT, while the posterior circulation collateral score was scored on baseline CT angiography by the core laboratory.

### Statistical Analysis

Baseline characteristics were presented using descriptive statistics. Dichotomous and ordinal parameters were compared using Pearson χ^2^ test or Fisher exact test. Continuous variables were tested using independent-samples *t* test or Mann-Whitney *U* test, after checking for the normality using histograms.

For the primary outcome, a multivariable ordinal logistics regression model was used to compare the use of IVT for a 1-step shift on the mRS score at 90 days follow-up. Continuous variables were checked on normality of distribution of the residuals using Q-Q plots. When no normality was seen, the variable was transformed using a natural logarithm. After exponentiating the regression coefficient, relative percentages were calculated using the following formula: (exponentiate [coefficient] −1)×100%. Adjusted odds ratios (aORs) or beta estimates with 95% CIs were used to present the regression model results.

All regression models were adjusted for potential confounders: age, sex, baseline National Institutes of Health Stroke Scale score, pre-mRS score (dichotomized 0–2 versus 3–5), diabetes, hypertension in patients’ history, systolic blood pressure when entering the hospital, the use of anticoagulation medication, the collaterals at CT angiography baseline, and the time between estimated LVO and groin puncture. These confounders were chosen based on univariable analyses complemented with parameters observed in previous literature and baseline differences. All analyses were performed using R (version 4.1.2.). A *P* value of <0.05 was defined as statistically significant for all analyses.

### Missing Values

Original data were used for the descriptive analyses, whereas multiple imputations with chained equitation were used for the missing data before conducting the regression analyses. The number of imputations was set at 50. The complete list of variables used for imputation is described in Supplemental S1.

### Subgroup Analyses

An interaction term was calculated to assess the interaction between occlusion location and IVT on the mRS score at 90 days. Exploratory subgroup analyses by occlusion location were performed. The same variables for adjustment were used as for the primary analysis, regardless of the group sizes.

## RESULTS

### Baseline Characteristics

**Figure 1.**
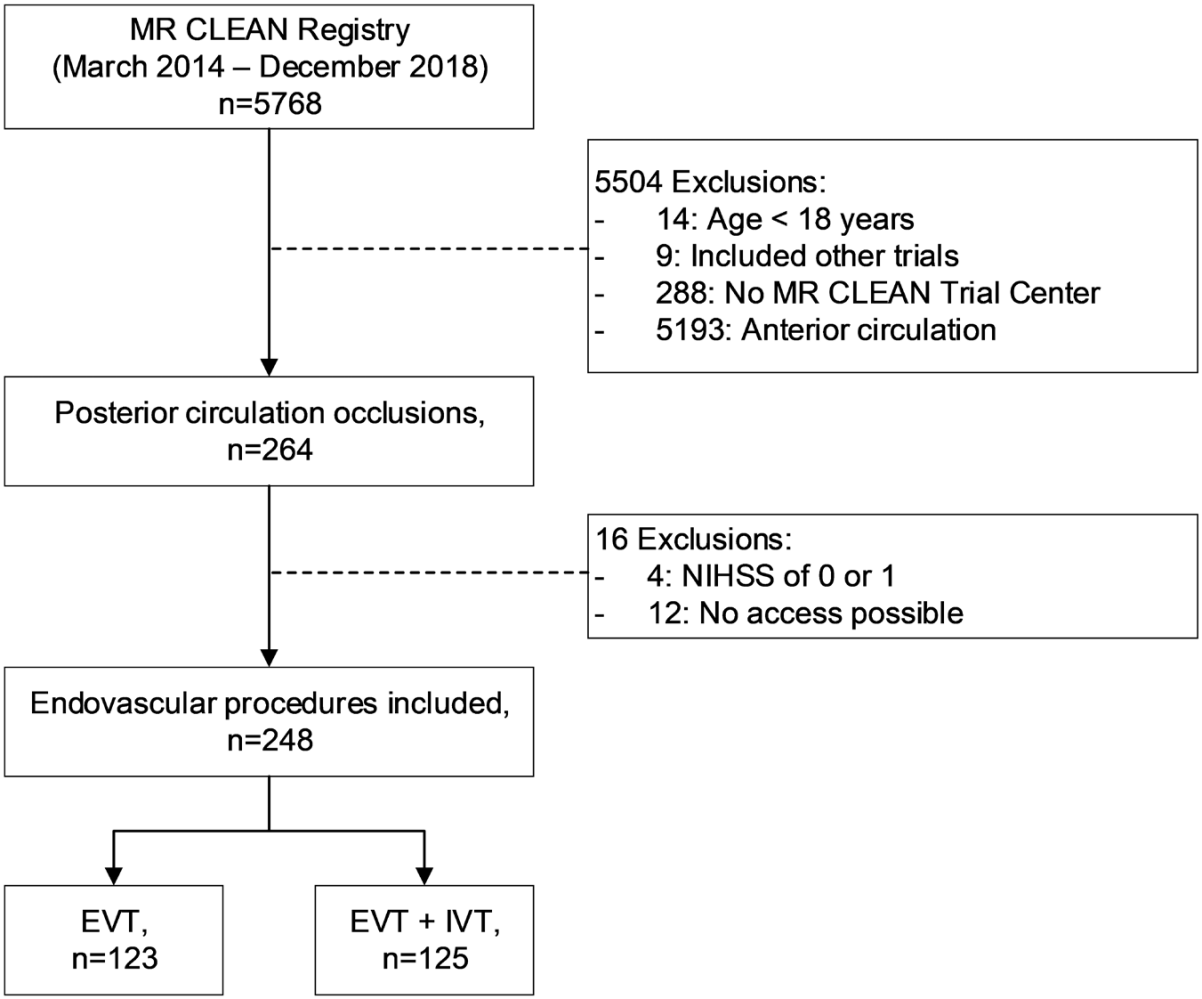
**Flow-chart of included patients in this study.** EVT indicates endovascular treatment; IVT, intravenous thrombolysis; MR CLEAN, Multicenter Randomized Clinical Trial of Endovascular Treatment for Acute Ischemic Stroke in the Netherlands; and NIHSS, National Institutes of Health Stroke Scale.

A total of 5768 patients were included in the MR CLEAN Registry, of which 264 patients had a posterior circulation occlusion. After applying the inclusion and exclusion criteria, a total of 248 patients were analyzed in the current study (Figure 1). Patients with IVT less often used anticoagulation before EVT, had lower pre-mRS scores, had faster onset to groin puncture times, and more often showed early recanalization compared with the patients treated without IVT (Table 1).

**Table 1.**
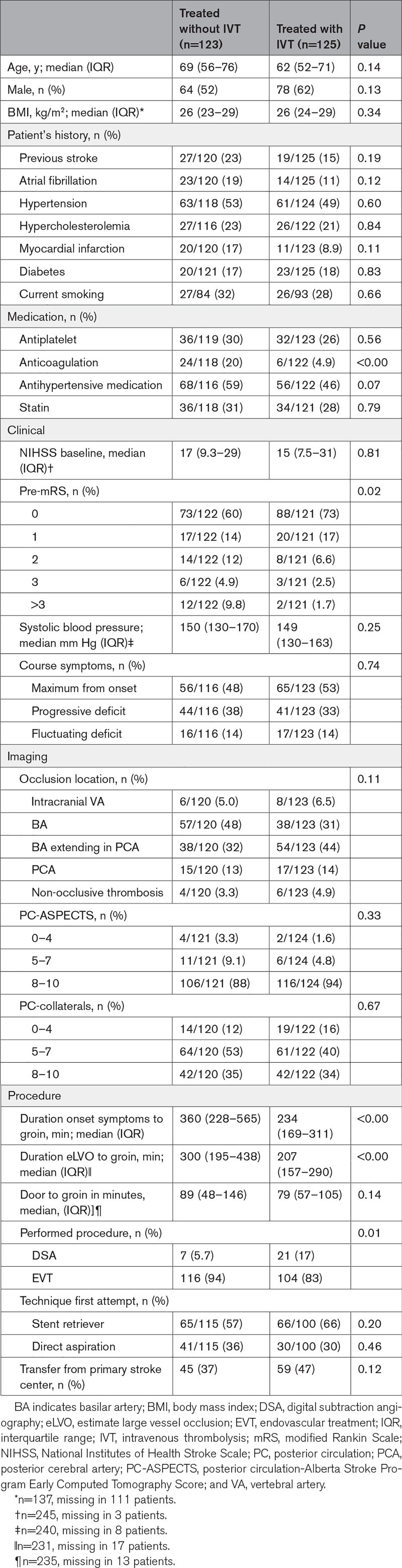
Baseline Characteristics of Included Patients

### Clinical Outcome

**Figure 2.**
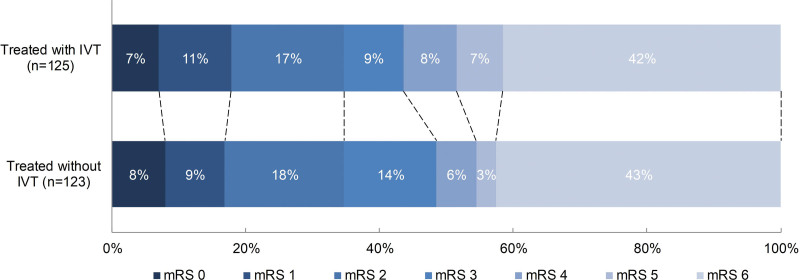
**Distribution of the modified Rankin Scale.** Multiple logistic regression with adjustment showed no significant difference between patients treated with intravenous thrombolysis (IVT) prior endovascular treatment (EVT) compared to patients treated with IVT prior EVT (adjusted common odds ratio, 1.04 [95%CI:0.61 – 1.76]). mRS indicates modified Rankin Scale.

There was no significant difference in the mRS score at 90 days between patients treated with IVT and without IVT (adjusted common odds ratio, 1.04 [95% CI, 0.61–1.76]; Figure 2). Also, no differences were seen in mortality and favorable functional outcome at 90 days, aOR, 0.93 (95% CI, 0.50–1.74), and aOR, 0.80 (95% CI, 0.43–1.49), respectively (Tables 2 and 3).

**Table 2.**
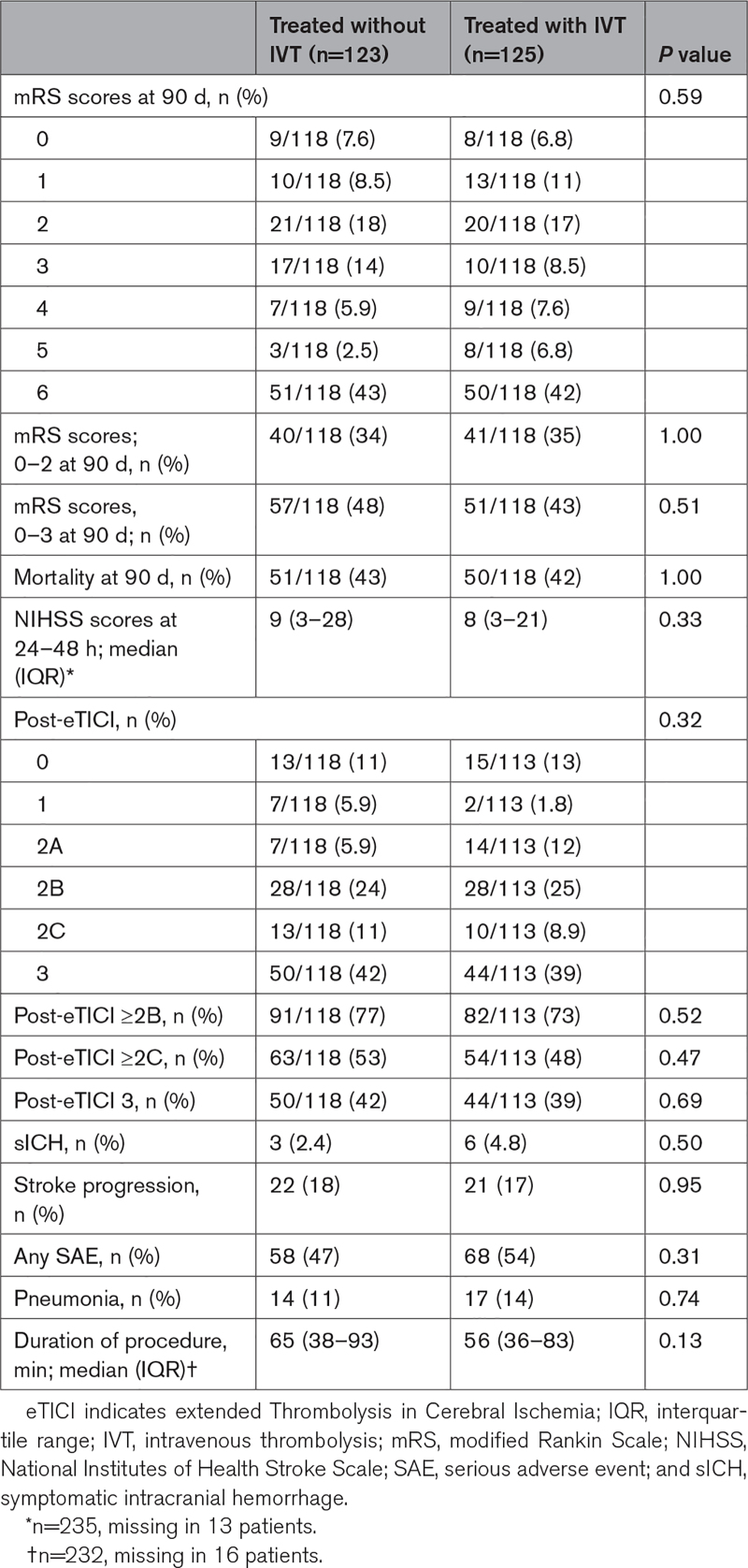
Outcomes Between Patients Treated With and Without IVT

**Table 3.**
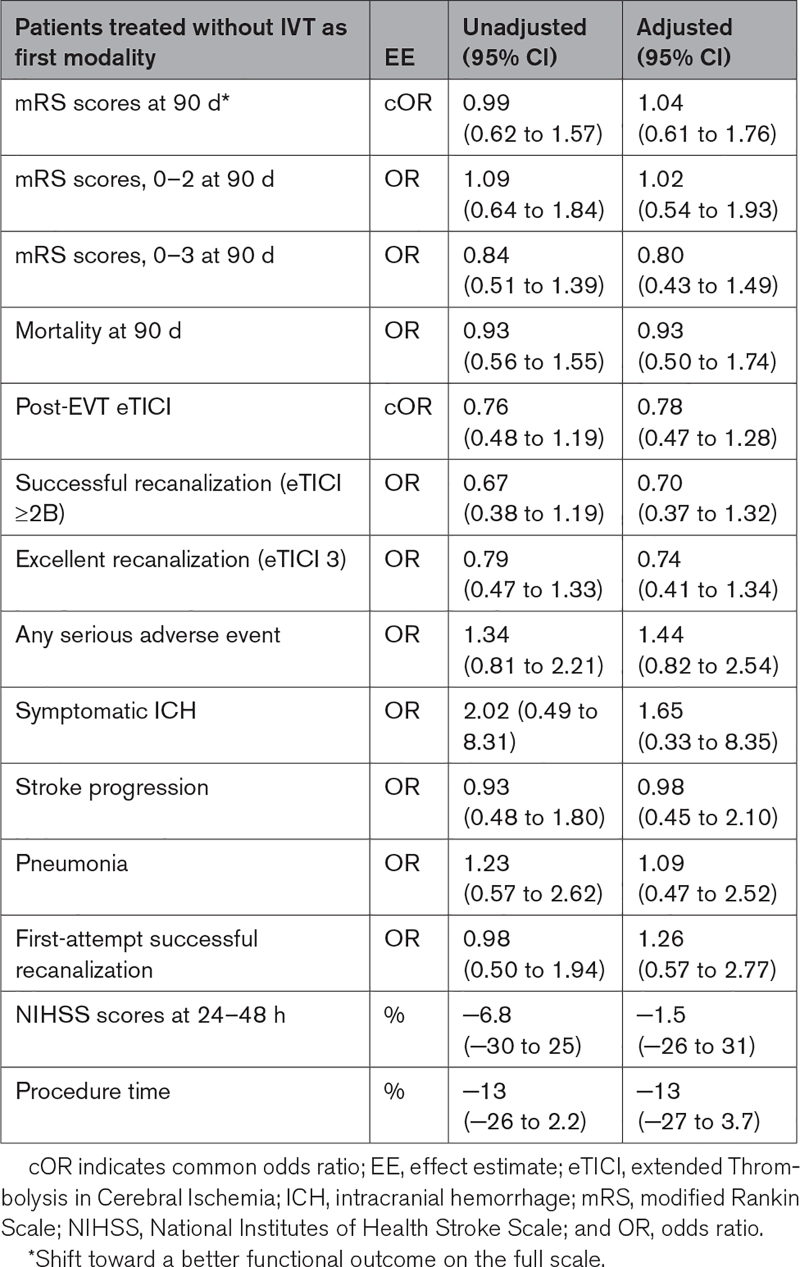
Outcomes of Regression Analyses on Clinical, Technical, and Safety Outcomes

### Technical Outcome

Although patients treated with IVT before EVT had an on average shorter procedure time (56 versus 65 minutes, *P*=0.13; Table 2), no significant differences were seen in the adjusted regression analysis after transforming the data (−13% [95% CI, −27 to 3.7]). Additionally, no differences were seen in first-attempt successful recanalization rates and successful recanalization rates (aOR, 1.26 [95% CI, 0.57–2.77 and aOR, 0.70 [95% CI, 0.37–1.32], respectively; Tables 2 and 3).

### Safety Outcome

In 47% of the patients treated without IVT and in 54% with IVT before EVT any SAE occurred (*P*=0.31). Symptomatic ICH was twice as often seen in patients treated with IVT before EVT (4.8% versus 2.4%) (Table 2); however, this difference was not statistically significant in regression analysis (aOR, 1.65 [95% CI, 0.33–8.35]; Table 3).

### Subgroup Analysis

There was a significant interaction between occlusion location and IVT on the mRS score at 90 days (*P*<0.00). In the subgroup analyses, IVT had a negative association with mRS score at 90 days (meaning higher mRS scores) in patients with an isolated posterior cerebral artery occlusion (adjusted common odds ratio, 0.08 [95% CI, 0.00–0.72]; Figure S1). There was no significant difference in favorable functional outcome in patients with isolated basilar artery occlusions treated with or without prior IVT (adjusted common odds ratio, 2.28 [95% CI, 0.95–5.49]).

## DISCUSSION

In this study, the use of IVT before EVT in patients with a posterior circulation occlusion did not lead to significant differences in clinical, technical, and safety outcomes.

Literature is scarce about the impact of IVT before EVT in patients with ischemic stroke due to posterior circulation occlusion. In the anterior circulation, multiple studies, including trials and registries, showed no superiority or noninferiority in patients treated with IVT before EVT on functional outcome at 90 days.^2,6,17^ No trials are performed yet on the effect of IVT before EVT in the posterior circulation.

In the BAOCHE, ATTENTION, and BEST trials 15%, 34%, and 27% of the patients received IVT, respectively,^9,11,12^ while 79% of patients in the BASICS trial received IVT.^10^ Main reason for the difference is the treatment window. BAOCHE included patients between 6 and 24 hours after symptom onset, the ATTENTION up to 12 hours of estimated time of BAO, and the BEST up to 8 hours after estimated time of BAO, while patients in the BASICS trial were included within 6 hours of estimated time of BAO. Another reason may be that BAOCHE, ATTENTION, and BEST included patients from China, where, to receive IVT, payment in advance is required.^9,11,12^ In the Netherlands, IVT is reimbursed, which may explain the higher rates of IVT in the MR CLEAN Registry.

The 4 above-mentioned trials showed ≈45% favorable functional outcome (mRS scores, 0–3) in the EVT group. Similar results are presented in the current study, Table S1 gives an overview of favorable functional outcome in patients with only a BAO. Favorable functional outcome was seen in 44% in patients with BAO treated with IVT and 42% in patients not treated with IVT. Despite the lower pre-mRS in patients treated with IVT, no differences in favorable functional outcome were seen.

Subgroup analysis on occlusion location (Figure S1) suggests that the potential benefit of IVT diminishes as the occlusion is more distally located when combined with EVT. It is known that IVT may more often lead to thrombus migration and, therefore, to more clot inaccessibility for thrombectomy.^18^ This may be a reason why the isolated patients with PCA are performing worse in our subgroup analysis. Additionally, treatment of PCA occlusion with EVT appears to be less established than treatment of BAO leading to a difference in treatment strategy which may have influenced outcome.^19^ IVT may have an effect on clot composition and size which may lead to easier clot extraction. Although review of the literature did not show fewer thrombectomy attempts when treated with IVT before EVT, 1 study suggested significantly reduction of thrombus size after IVT.^20^ In combination with a more aggressive treatment approach in patients with BAO, this could substantiate our subgroup analyses. However, analyses were performed on a limited number of patients with the same adjustments as for our primary analysis precluding strong conclusions, indicating the need for pooling data with other similar studies to increase power.

In 4.8% of the patients treated with IVT before EVT a sICH was seen. Comparable sICH rates were seen in the EVT groups of the BASICS (4.5%), ATTENTION (6%), BEST (8%), and BAOCHE (5%) trials.^9–12^ However, these EVT groups include patients treated with and without IVT before EVT, while different sICH criteria were used. IVT in combination with EVT could potentially increase the rates of sICH compared with EVT alone, especially because of the concomitant periprocedural use of anticoagulation potential vessel wall damage due to the thrombectomy materials. However, we did not observe this in our results. Explanations are at the moment purely hypothetical. Literature suggests better collateral circulation in the posterior circulation compared with the anterior circulation as a possible explanation for the lower sICH rates.^13^

One reason to start EVT directly, before administering IVT, is because of the potential time delay. Especially when patients are directly presented at a stroke center. However, another MR CLEAN Registry study did not show a delay in door-to-groin times when IVT was given before EVT.^21^ Additionally, patients treated with IVT before EVT showed higher rates of early recanalization (17%) compared with patients treated without IVT before EVT (5.7%). These higher rates did not lead to differences in clinical outcome. The clinical outcome measure (mRS score at 90 days) may not be optimal to detect small differences in clinical outcomes, and a more sensitive outcome measure may be needed.

Our study has limitations. First of all, patients who recanalized after treatment with IVT alone were not included in the MR CLEAN Registry, potentially causing an underestimation of the effect of IVT before EVT in patients with a BAO. Furthermore, we excluded patients (n=12) in whom no intracranial access was obtained with thrombectomy materials. Second, during the MR CLEAN Registry, many patients with basilar artery occlusion were included (when eligible) in the BASICS trial, causing also potential selection bias. However, this selection bias was probably limited, since a previous publication showed similar favorable functional outcome in patients treated within the MR CLEAN Registry compared with the BASICS trial.^22^ Third, our registry based on real-world data has the limitations of a nonrandomized study: use of IVT was left to the treating physician and the numbers are small. In the American Heart Association guidelines IVT is contraindicated in some patients using anticoagulation and with high systolic blood pressures^23^; to minimize this effect analyses were adjusted for these potential confounders. Finally, in this study, thrombus characteristics, such as the length of the occlusion and thrombus density, were not taken into account in the analysis. However, the impact of these characteristics seems to be limited.^24^

## CONCLUSIONS

We found no significant differences in clinical, technical, and safety outcomes between patients with an LVO in the posterior circulation treated with or without IVT before endovascular therapy. Our results are in line with the literature about the anterior circulation.

## ARTICLE INFORMATION

### Acknowledgments

The authors thank all the investigators of the MR CLEAN Registry (Multicenter Randomized Controlled Trial of Endovascular Treatment for Acute Ischemic Stroke in the Netherlands) for their effort and contributions.

### Sources of Funding

The MR CLEAN Registry (Multicenter Randomized Clinical Trial of Endovascular Treatment of Acute Ischemic Stroke) was partly funded by Stichting Toegepast Wetenschappelijk Instituut voor Neuromodulatie, Erasmus MC University Medical Center, Maastricht University Medical Center, and Amsterdam University Medical Center.

### Disclosures

Dr Majoie reports grants from the Netherlands Cardiovascular Research Initiative, an initiative of the Dutch Heart Foundation, European Commission, Healthcare Evaluation Netherlands, Stichting Toegepast Wetenschappelijk Instituut voor Neuromodulatie Foundation, and Stryker (all paid to institution); and is a (minority interest) shareholder of Nicolab. Dr Emmer reports grants from leading the Change Healthcare Evaluation program, ZorgOnderzoek Nederland Medische Wetenschappen (ZonMw), Nico-Lab, and Top Consortia for Knowledge and Innovation- Public Private Partnership (TKI-PPP) Grant Topsector Lifesciences (all paid to institution); and participates as a representative of the European Union of Medical Specialists (UEMS) Neuroradiology Dutch, and as Board member of the Dutch Society of Radiology. Dr Vos reports compensation from Stryker Corporation for other services. Dr van Doormaal reports consulting fees from Stryker, Siemens, and Stryker (all paid to institution); participates in the advisory board of DX Medical solutions; and is shareholder of DX Medical Solutions. Dr Yoo reports grants from Medtronic, Cerenovus, Penumbra, Stryker, Genentech, and Johnson & Johnson Medical Devices & Diagnostics Group, Latin America LLC; holds stock options in Nico-Lab, Insera; and is a consultant for Vesalio, Cerenovus, Rapid Medical Ltd, Penumbra, Inc, Nico-Lab, ZOLL Circulation, Inc, and Philips, all outside the submitted work; compensation from American Heart Association for other services; compensation from National Institutes of Health for data and safety monitoring services. Dr van Zwam reports speaker fees from Stryker, Cerenovus, and Nicolab, and consulting fees from Philips (all paid to institution); participated in the advisory boards of WeTrust (Philips) and ANAIS ([Advanced Neurovascular Access in Combination With a Stent Retriever in Patients With Acute Ischemic Stroke]; Anaconda; all paid to institution); and participated in the advisory boards of InEcxtremis (CHU Montpellier, Montpellier, France) and DISTAL ([Endovascular Therapy Plus Best Medical Treatment (BMT) Versus BMT Alone for Medium Vessel Occlusion Stroke - a Pragmatic, International, Multicentre, Randomized Trial]; University Hospital Basel, Basel, Switzerland), studies for which no payments were received. The other authors report no conflicts.

### Supplemental Material

Supplemental S1

Table S1

Figure S1

## APPENDIX

MR CLEAN Registry Investigators

Executive Committee

Diederik W.J. Dippel (Department of Neurology, Erasmus MC University Medical Center), Aad van der Lugt (Department of Radiology, Erasmus MC University Medical Center), Charles B.L.M. Majoie (Department of Radiology and Nuclear Medicine, Amsterdam UMC, University of Amsterdam), Yvo B.W.E.M. Roos (Department of Neurology, Amsterdam UMC, University of Amsterdam, Amsterdam), Robert J. van Oostenbrugge; (Department of Neurology, Maastricht University Medical Center and Cardiovascular Research Institute Maastricht (CARIM), Wim H. van Zwam (Department of Radiology, Maastricht University Medical Center and Cardiovascular Research Institute Maastricht [CARIM]), Jelis Boiten (Department of Neurology, Haaglanden MC, the Hague), Jan Albert Vos (Department of Radiology, St. Antonius Hospital, Nieuwegein).

Study Coordinators

Ivo G.H. Jansen (Department of Radiology and Nuclear Medicine, Amsterdam UMC, University of Amsterdam), Maxim J.H.L. Mulder (Departments of Neurology and Radiology, Erasmus MC University Medical Center). Robert-Jan B. Goldhoorn (Department of Neurology, and Department of Radiology, Maastricht University Medical Center and Cardiovascular Research Institute Maastricht (CARIM), Kars C.J. Compagne (Department of Radiology, Erasmus MC University Medical Center); Manon Kappelhof (Department of Radiology and Nuclear Medicine, Amsterdam UMC, University of Amsterdam, Amsterdam), Josje Brouwer (Department of Neurology, Amsterdam UMC, University of Amsterdam, Amsterdam Sanne J. den Hartog (Departments of Neurology, Radiology, and Public Health, Erasmus MC University Medical Center), Wouter H. Hinsenveld (Departments of Neurology and Radiology, Maastricht University Medical Center and Cardiovascular Research Institute Maastricht (CARIM).

Local Principal Investigators

Diederik W.J. Dippel (Department of Neurology, Erasmus MC University Medical Center), Bob Roozenbeek (Department of Neurology, Erasmus MC University Medical Center), Aad van der Lugt (Department of Radiology, Erasmus MC University Medical Center), Charles B.L.M. Majoie (Department of Radiology and Nuclear Medicine, Amsterdam UMC, University of Amsterdam, Amsterdam), Yvo B.W.E.M. Roos (Department of Neurology, Amsterdam UMC, University of Amsterdam, Amsterdam), Bart J. Emmer (Department of Radiology and Nuclear Medicine, Amsterdam UMC, University of Amsterdam, Amsterdam), Jonathan M. Coutinho (Department of Neurology, Amsterdam UMC, University of Amsterdam, Amsterdam), Wouter J. Schonewille (Department of Neurology, St. Antonius Hospital, Nieuwegein), Jan Albert Vos (Department of Radiology, St. Antonius Hospital, Nieuwegein), Marieke J.H. Wermer (Department of Neurology, Leiden University Medical Center), Marianne A.A. van Walderveen (Department of Radiology, Leiden University Medical Center), Adriaan C.G.M. van Es (Department of Radiology, Leiden University Medical Center), Julie Staals (Department of Neurology, Maastricht University Medical Center and Cardiovascular Research Institute Maastricht [CARIM]), Robert J. van Oostenbrugge (Department of Neurology, Maastricht University Medical Center and Cardiovascular Research Institute Maastricht [CARIM]), Wim H. van Zwam (Department of Radiology, Maastricht University Medical Center and Cardiovascular Research Institute Maastricht [CARIM]), Jeannette Hofmeijer (Department of Neurology, Rijnstate Hospital, Arnhem), Jasper M. Martens (Department of Radiology, Rijnstate Hospital, Arnhem), Geert J. Lycklama à Nijeholt (Department of Radiology, Haaglanden MC, the Hague), Jelis Boiten (Department of Neurology, Haaglanden MC, the Hague), Sebastiaan F. de Bruijn (Department of Neurology, HAGA Hospital, the Hague), Lukas C. van Dijk (Department of Radiology, HAGA Hospital, the Hague), H. Bart van der Worp (Department of Neurology, University Medical Center Utrecht), Rob H. Lo (Department of Radiology, University Medical Center Utrecht), Ewoud J. van Dijk (Department of Neurology, Radboud University Medical Center, Nijmegen), Hieronymus D. Boogaarts (Department of Neurosurgery, Radboud University Medical Center, Nijmegen), J. de Vries (Department of Neurology, Isala Klinieken, Zwolle), Paul L.M. de Kort (Department of Neurology, Elisabeth-TweeSteden ziekenhuis, Tilburg), Julia van Tuijl (Department of Neurology, Elisabeth-TweeSteden ziekenhuis, Tilburg), Jo P. Peluso (Department of Radiology, Elisabeth-TweeSteden ziekenhuis, Tilburg), Puck Fransen (Department of Neurology, Isala Klinieken, Zwolle), Jan S.P. van den Berg (Department of Neurology, Isala Klinieken, Zwolle), Boudewijn A.A.M. van Hasselt (Department of Radiology, Isala Klinieken, Zwolle), Leo A.M. Aerden (Department of Neurology, Reinier de Graaf Gasthuis, Delft), René J. Dallinga (Department of Radiology, Reinier de Graaf Gasthuis, Delft), Maarten Uyttenboogaart (Department of Neurology, University Medical Center Groningen), Omid Eschgi (Department of Radiology, University Medical Center Groningen), Reinoud P.H. Bokkers (Department of Radiology, University Medical Center Groningen), Tobien H.C.M.L. Schreuder (Department of Neurology, Atrium Medical Center, Heerlen), Roel J.J. Heijboer (Department of Radiology, Atrium Medical Center, Heerlen), Koos Keizer (Department of Neurology, Catharina Hospital, Eindhoven), Lonneke S.F. Yo (Department of Radiology, Catharina Hospital, Eindhoven), Heleen M. den Hertog (Department of Neurology, Isala Klinieken, Zwolle), Emiel J.C. Sturm (Department of Neurology, Medisch Spectrum Twente, Enschede) Paul J.A.M. Brouwers (Department of Neurology, Medisch Spectrum Twente, Enschede).

Imaging Assessment Committee

Charles B.L.M. Majoie (chair; Department of Radiology and Nuclear Medicine, Amsterdam UMC, University of Amsterdam), Wim H. van Zwam (Department of Radiology, Maastricht University Medical Center and Cardiovascular Research Institute Maastricht [CARIM]), Aad van der Lugt (Department of Radiology, Erasmus MC University Medical Center), Geert J. Lycklama à Nijeholt (Department of Radiology, Haaglanden MC, the Hague), Marianne A.A. van Walderveen (Department of Radiology, Leiden University Medical Center), Marieke E.S. Sprengers (Department of Radiology and Nuclear Medicine, Amsterdam UMC, University of Amsterdam) Sjoerd F.M. Jenniskens (Department of Radiology, Radboud University Medical Center, Nijmegen), René van den Berg (Department of Radiology and Nuclear Medicine, Amsterdam UMC, University of Amsterdam), Albert J. Yoo (Department of Radiology, Texas Stroke Institute), Ludo F.M. Beenen (Department of Radiology and Nuclear Medicine, Amsterdam UMC, University of Amsterdam), Alida A. Postma (Department of Radiology, Maastricht University Medical Center and Cardiovascular Research Institute Maastricht [CARIM]), Stefan D. Roosendaal (Department of Radiology and Nuclear Medicine, Amsterdam UMC, University of Amsterdam), Bas F.W. van der Kallen (Department of Radiology, Haaglanden MC, the Hague), Ido R. van den Wijngaard (Department of Radiology, Haaglanden MC, the Hague), Adriaan C.G.M. van Es (Department of Radiology, Leiden University Medical Center), Bart J. Emmer (Department of Radiology and Nuclear Medicine, Amsterdam UMC, University of Amsterdam), Jasper M. Martens (Department of Radiology, Rijnstate Hospital, Arnhem), Lonneke S.F. Yo (Department of Radiology, Catharina Hospital, Eindhoven), Jan Albert Vos (Department of Radiology, St. Antonius Hospital, Nieuwegein), Joost Bot (Department of Radiology, Amsterdam UMC, Vrije Universiteit van Amsterdam, Amsterdam), Pieter-Jan van Doormaal (Department of Radiology, Erasmus MC University Medical Center), Anton Meijer (Department of Radiology, Radboud University Medical Center, Nijmegen), Elyas Ghariq (Department of Radiology, Haaglanden MC, the Hague), Reinoud P.H. Bokkers (Department of Radiology, University Medical Center Groningen), Marc P. van Proosdij (Department of Radiology, Noordwest Ziekenhuisgroep, Alkmaar), G. Menno Krietemeijer (Department of Radiology, Catharina Hospital, Eindhoven), Jo P. Peluso (Department of Radiology, Elisabeth-TweeSteden ziekenhuis, Tilburg), Hieronymus D. Boogaarts (Department of Neurosurgery, Radboud University Medical Center, Nijmegen), Rob Lo (Department of Radiology, University Medical Center Utrecht), Wouter Dinkelaar (Department of Radiology, Albert Schweitzer Hospital, Dordrecht), Auke P.A. Appelman (Department of Radiology, University Medical Center Groningen), Bas Hammer (Department of Radiology, HAGA Hospital, the Hague), Sjoert Pegge (Department of Neurology Radiology, Radboud University Medical Center, Nijmegen), Anouk van der Hoorn (Department of Radiology, University Medical Center Groningen), Saman Vinke (Department of Neurosurgery, Radboud University Medical Center, Nijmegen), Sandra Cornelissen (Department of Radiology, Erasmus MC University Medical Center), Christiaan van der Leij (Department of Radiology, Maastricht University Medical Center and Cardiovascular Research Institute Maastricht [CARIM]), Rutger Brans (Department of Radiology, Maastricht University Medical Center and Cardiovascular Research Institute Maastricht [CARIM]).

Writing Committee

Diederik W.J. Dippel (chair) (Department of Neurology, Erasmus MC University Medical Center), Aad van der Lugt (Department of Radiology, Erasmus MC University Medical Center), Charles B.L.M. Majoie (Department of Radiology and Nuclear Medicine, Amsterdam UMC, University of Amsterdam), Yvo B.W.E.M. Roos (Department of Neurology, Amsterdam UMC, University of Amsterdam), Robert J. van Oostenbrugge (Department of Neurology, Maastricht University Medical Center and Cardiovascular Research Institute Maastricht [CARIM]), Wim H. van Zwam (Department of Radiology, Maastricht University Medical Center and Cardiovascular Research Institute Maastricht [CARIM]), Geert J. Lycklama à Nijeholt (Department of Radiology, Haaglanden MC, the Hague), Jelis Boiten (Department of Neurology, Haaglanden MC, the Hague), Jan Albert Vos (Department of Radiology, St. Antonius Hospital, Nieuwegein), Wouter J. Schonewille (Department of Neurology, St. Antonius Hospital, Nieuwegein), Jeannette Hofmeijer (Department of Neurology, Rijnstate Hospital, Arnhem), Jasper M. Martens (Department of Radiology, Rijnstate Hospital, Arnhem), H. Bart van der Worp (Department of Neurology, University Medical Center Utrecht), Rob H. Lo (Department of Radiology, University Medical Center Utrecht).

Adverse Event Committee

Robert J. van Oostenbrugge (chair; Department of Neurology, Maastricht University Medical Center and Cardiovascular Research Institute Maastricht [CARIM]), Jeannette Hofmeijer (Department of Radiology, Rijnstate Hospital, Arnhem) H. Zwenneke Flach (Department of Radiology, Isala Klinieken, Zwolle).

Trial Methodologist

Hester F. Lingsma (Department of Public Health, Erasmus MC University Medical Center).

Research Nurses/Local Trial Coordinators

Naziha el Ghannouti (Department of Neurology, Erasmus MC University Medical Center); Martin Sterrenberg (Department of Neurology, Erasmus MC University Medical Center), Wilma Pellikaan (Department of Neurology, St. Antonius Hospital, Nieuwegein), Rita Sprengers (Department of Neurology, Amsterdam UMC, University of Amsterdam), Marjan Elfrink (Department of Neurology, Rijnstate Hospital, Arnhem), Michelle Simons (Department of Neurology, Rijnstate Hospital, Arnhem), Marjolein Vossers (Department of Radiology, Rijnstate Hospital, Arnhem), Joke de Meris (Department of Neurology, Haaglanden MC, the Hague), Tamara Vermeulen (Department of Neurology, Haaglanden MC, the Hague), Annet Geerlings (Department of Neurology, Radboud University Medical Center, Nijmegen), Gina van Vemde (Department of Neurology, Isala Klinieken, Zwolle), Tiny Simons (Department of Neurology, Atrium Medical Center, Heerlen), Gert Messchendorp (Department of Neurology, University Medical Center Groningen), Nynke Nicolaij (Department of Neurology, University Medical Center Groningen), Hester Bongenaar (Department of Neurology, Catharina Hospital, Eindhoven), Karin Bodde Department of Neurology, Reinier de Graaf Gasthuis, Delft Sandra Kleijn (Department of Neurology, Medisch Spectrum Twente, Enschede), Jasmijn Lodico (Department of Neurology, Medisch Spectrum Twente, Enschede), Hanneke Droste (Department of Neurology, Medisch Spectrum Twente, Enschede), Maureen Wollaert (Department of Neurology, Maastricht University Medical Center and Cardiovascular Research Institute Maastricht [CARIM]), Sabrina Verheesen (Department of Neurology, Maastricht University Medical Center and Cardiovascular Research Institute Maastricht [CARIM]), D. Jeurrissen (Department of Neurology, Maastricht University Medical Center and Cardiovascular Research Institute Maastricht [CARIM]), Erna Bos (Department of Neurology, Leiden University Medical Center), Yvonne Drabbe (Department of Neurology, HAGA Hospital, the Hague), Michelle Sandiman (Department of Neurology, HAGA Hospital, the Hague), Nicoline Aaldering (Department of Neurology, Rijnstate Hospital, Arnhem), Berber Zweedijk (Department of Neurology, University Medical Center Utrecht), Jocova Vervoort (Department of Neurology, Elisabeth-TweeSteden ziekenhuis, Tilburg), Eva Ponjee (Department of Neurology, Isala Klinieken, Zwolle) Sharon Romviel (Department of Neurology, Radboud University Medical Center, Nijmegen), Karin Kanselaar (Department of Neurology, Radboud University Medical Center, Nijmegen), Denn Barning (Department of Radiology, Leiden University Medical Center),

Clinical/Imaging Data Acquisition

Esmee Venema (Department of Public Health, Erasmus MC University Medical Center) Vicky Chalos (Departments of Neurology and Public Health, Erasmus MC University Medical Center) Ralph R. Geuskens (Department of Radiology and Nuclear Medicine, Amsterdam UMC, University of Amsterdam), Tim van Straaten (Department of Neurology, Radboud University Medical Center, Nijmegen), Saliha Ergezen (Department of Neurology, Erasmus MC University Medical Center), Roger R.M. Harmsma (Department of Neurology, Erasmus MC University Medical Center), Daan Muijres; (Department of Neurology, Erasmus MC University Medical Center) Anouk de Jong (Department of Neurology, Erasmus MC University Medical Center), Olvert A. Berkhemer (Department of Neurology, Erasmus MC University Medical Center; Department of Radiology and Nuclear Medicine, Amsterdam UMC, University of Amsterdam; Department of Radiology, Maastricht University Medical Center and Cardiovascular Research Institute Maastricht [CARIM]), Anna M.M. Boers (Department of Radiology and Nuclear Medicine, Amsterdam UMC, University of Amsterdam) J. Huguet (Department of Radiology and Nuclear Medicine, Amsterdam UMC, University of Amsterdam), P.F.C. Groot (Department of Radiology and Nuclear Medicine, Amsterdam UMC, University of Amsterdam), Marieke A. Mens (Department of Radiology and Nuclear Medicine, Amsterdam UMC, University of Amsterdam), Katinka R. van Kranendonk (Department of Radiology and Nuclear Medicine, Amsterdam UMC, University of Amsterdam), Kilian M. Treurniet (Department of Radiology and Nuclear Medicine, Amsterdam UMC, University of Amsterdam), Manon L. Tolhuisen (Department of Radiology and Nuclear Medicine, Biomedical Engineering & Physics, Amsterdam UMC, University of Amsterdam), Heitor Alves (Department of Radiology and Nuclear Medicine, Amsterdam UMC, University of Amsterdam), Annick J. Weterings (Department of Radiology and Nuclear Medicine, Amsterdam UMC, University of Amsterdam), Eleonora L.F. Kirkels (Department of Radiology and Nuclear Medicine, Amsterdam UMC, University of Amsterdam), Eva J.H.F. Voogd (Department of Neurology, Rijnstate Hospital, Arnhem), Lieve M. Schupp (Department of Radiology and Nuclear Medicine, Amsterdam UMC, University of Amsterdam), Sabine L. Collette (Departments of Neurology and Radiology, University Medical Center Groningen), Adrien E.D. Groot (Department of Neurology, Amsterdam UMC, University of Amsterdam, Amsterdam), Natalie E. LeCouffe (Department of Neurology, Amsterdam UMC, University of Amsterdam, Amsterdam), Praneeta R. Konduri (Department of Biomedical Engineering & Physics, Amsterdam UMC, University of Amsterdam), Haryadi Prasetya (Department of Biomedical Engineering & Physics, Amsterdam UMC, University of Amsterdam), Nerea Arrarte-Terreros (Department of Biomedical Engineering & Physics, Amsterdam UMC, University of Amsterdam), Lucas A. Ramos (Department of Biomedical Engineering & Physics, Amsterdam UMC, University of Amsterdam), Nikki Boodt (Departments of Neurology, Radiology, and Public Health, Erasmus MC University Medical Center), F. Anne V. Pirson (Department of Neurology, Maastricht University Medical Center and Cardiovascular Research Institute Maastricht [CARIM]), Agnetha A.E. Bruggeman (Department of Radiology and Nuclear Medicine, Amsterdam UMC, University of Amsterdam).

## Supplementary Material


