# Supplementary material for: Intravenous Thrombolysis Before Endovascular Treatment in Posterior Circulation Occlusions: A MR CLEAN Registry Study
Source: Stroke. 2024 Jan 4;55(2):403–12. doi: 10.1161/STROKEAHA.123.043777 (PMC10802980; doi:10.1161/STROKEAHA.123.043777)
Supplement: Supplementary file 1 [file str-55-403-s001.pdf]

SUPPLEMENTAL MATERIAL

Supplemental S1. List of used variables as predictors in multiple imputations

Pre-TICI score, post-TICI score, transferred from primary stroke hospital, previous stroke, myocardial infarction, peripheral arterial disease, diabetes mellitus, hypertension, atrial fibrillation, hypercholesterolemia, current smoking, use of antiplatelet, use of antihypertensive, use of anticoagulant, use of statins, systolic blood pressure, pre-mRS, performed procedure, mRS score at 90 days, NIHSS at 24-48 hours, age, sex, NIHSS at baseline, total attempts, mRS reversed score, PC-ASPECTS at baseline, location of occlusions, collaterals score at CTA, time between estimated large vessel occlusion and groin puncture, time between enter hospital and groin puncture, duration of procedure, BMI, time between estimated large vessel occlusion and last seen well, onset time last seen well, the occurrence of any serious adverse event.

Table S1. Overview of patients with a basilar artery occlusion with favorable functional outcome

	Treated without IVT (n=95)		Treated with IVT (n=92)	
mRS 0-3 at 90 days – n/N (%)	40/95	(44)	37/92	(42)

mRS, modified Rankin Scale; IVT, intravenous thrombolysis.

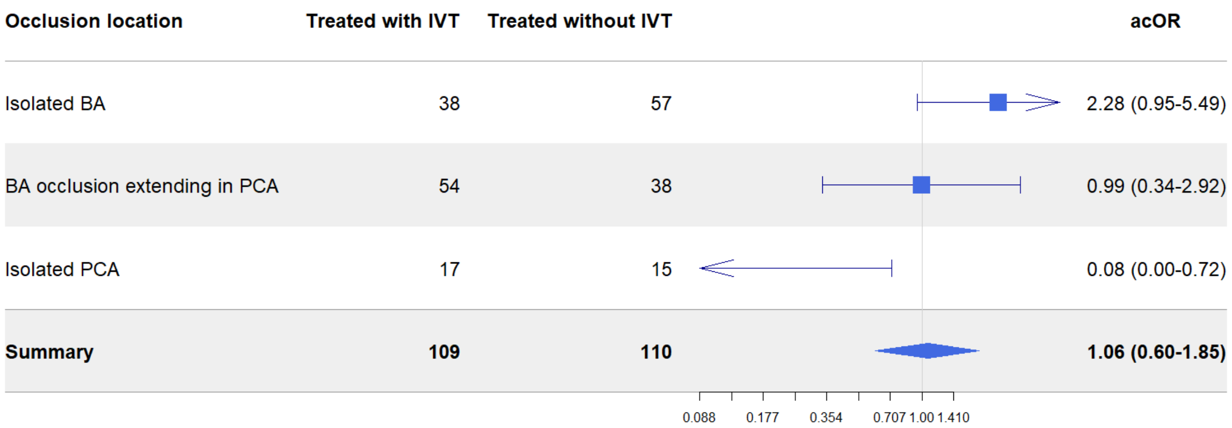

Figure S1. Subgroup analyses between patients treated with IVT prior to EVT compared to patients treated without IVT prior to EVT on mRS score at 90 days follow-up (shift towards a better functional outcome on the full scale) by occlusion location.

IVT, intravenous thrombolysis; BA, basilar artery; PCA, posterior cerebral artery.
