# Supplementary material for: Intravenous Thrombolysis Before Endovascular Treatment in Posterior Circulation Occlusions: A MR CLEAN Registry Study
Source: Stroke. 2024 Jan 4;55(2):403–12. doi: 10.1161/STROKEAHA.123.043777 (PMC10802980; doi:10.1161/STROKEAHA.123.043777)
Supplement: Supplementary file 2 [file str-55-403-s002.pdf]

STROBE Statement—Checklist of items that should be included in reports of ***cohort studies***

	Item No	Recommendation
**Title and abstract**	1	(*a*) Indicate the study's design with a commonly used term in the title or the abstract<br>*Title: Registry*<br>*Abstract: nationwide, prospective, multicenter registry*
		(*b*) Provide in the abstract an informative and balanced summary of what was done and what was found<br>*Page 4: abstract.*
**Introduction**		
Background/rationale	2	Explain the scientific background and rationale for the investigation being reported<br>*Literature about intravenous thrombolysis prior to endovascular treatment in the posterior circulation is limited. No RCT's are performed.*
Objectives	3	State specific objectives, including any prespecified hypotheses<br>*We aimed to investigate the clinical, safety, and technical outcomes of patients with a posterior large vessel occlusion treated with and without intravenous thrombolysis prior to endovascular treatment.*
**Methods**		
Study design	4	Present key elements of study design early in the paper<br>*Patients were included from the MR CLEAN (Multicenter Randomized Clinical Trial of Endovascular Treatment for Acute Ischemic Stroke in the Netherlands) Registry: a prospective, observational study in 18 EVT performing centers in the Netherlands.*
Setting	5	Describe the setting, locations, and relevant dates, including periods of recruitment, exposure, follow-up, and data collection<br>*MR CLEAN Registry included patients between March 2014 and December 2018. The primary outcome (modified Rankin Scale) was scored at 90 days as part of standard medical care.*
Participants	6	(*a*) Give the eligibility criteria, and the sources and methods of selection of participants. Describe methods of follow-up<br>*The following inclusion criteria were used: age ≥ 18 years, NIHSS ≥ 2, occlusion in the posterior circulation confirmed by CT-angiography. Patients in whom no intracranial access was obtained were excluded.*
		(*b*) For matched studies, give matching criteria and number of exposed and unexposed
Variables	7	Clearly define all outcomes, exposures, predictors, potential confounders, and effect modifiers. Give diagnostic criteria, if applicable<br>*Described in section: "Outcomes measures," "Imaging assessment" and "Statistical analysis."*
Data sources/ measurement	8*	For each variable of interest, give sources of data and details of methods of assessment (measurement). Describe comparability of assessment methods if there is more than one group<br>*Described in section: "Outcomes measures" and "Imaging assessment."*
Bias	9	Describe any efforts to address potential sources of bias<br>*Statistical analysis: All regression models were adjusted for potential confounders: age, sex, baseline NIHSS score, pre-mRS score, diabetes mellitus, hypertension in patients' history, systolic blood pressure when entering the hospital, the use of anticoagulation medication, the collaterals at CTA baseline, and the time between estimated large vessel occlusion and groin puncture.*
Study size	10	Explain how the study size was arrived at<br>*Described in Results and flow-chart. Out of the 5768 patients included in the MR*

		CLEAN Registry, 264 patients were treated for posterior large vessel occlusion stroke. 248 patients are included in the analysis after applying the in- and exclusion criteria.
Quantitative variables	11	Explain how quantitative variables were handled in the analyses. If applicable, describe which groupings were chosen and why<br>*Described in statistical analysis: Baseline characteristics were presented using descriptive statistic and are indicated in the tables.*
Statistical methods	12	(*a*) Describe all statistical methods, including those used to control for confounding<br>*Described in detail in "Statistical analysis." For the primary outcome, a multivariable ordinal logistics regression model was used to compare the use of IVT for a one-step shift on the mRS score at 90 days follow-up. Adjusted odds ratios or beta estimates with 95% confidence intervals were used to present the regression model results.*
		(*b*) Describe any methods used to examine subgroups and interactions<br>*Described in "Subgroup analyses." An interaction terms was calculated to assess the interaction between occlusion location and IVT on the mRS score at 90 days. Same variables for adjustment were used as for the primary analysis.*
		(*c*) Explain how missing data were addressed<br>*Described in "Missing values" and "Supplemental 1." Multiple imputations were used for the missing data. Original data were used for the descriptive analyses. RStudio (version 1.3.1093) was used for all analyses.*
		(*d*) If applicable, explain how loss to follow-up was addressed<br>*The excluded patients are presented in the flow-chart (figure 1).*
		(*e*) Describe any sensitivity analyses<br>*NA*
**Results**		
Participants	13*	(a) Report numbers of individuals at each stage of study—eg numbers potentially eligible, examined for eligibility, confirmed eligible, included in the study, completing follow-up, and analysed<br>*A total of 5768 patients were included in the MR CLEAN Registry. After applying the in- and exclusion criteria, a total of 248 patients were analysed in the current study (Figure 1).*
		(b) Give reasons for non-participation at each stage<br>*NA*
		(c) Consider use of a flow diagram<br>*See figure 1.*
Descriptive data	14*	(a) Give characteristics of study participants (eg demographic, clinical, social) and information on exposures and potential confounders<br>*Described in "Baseline characteristics" and in table 1. Patients with IVT less often used anticoagulation prior to EVT, had lower pre-mRS scores, had faster onset to groin puncture times and more often showed early recanalization compared to the patients treated without IVT.*
		(b) Indicate number of participants with missing data for each variable of interest<br>*Indicated in table 1 and 2.*
		(c) Summarise follow-up time (eg, average and total amount)<br>*NA*
Outcome data	15*	Report numbers of outcome events or summary measures over time<br>*The primary and secondary outcomes are provided in table 2 and figure 2.*
Main results	16	(*a*) Give unadjusted estimates and, if applicable, confounder-adjusted estimates and

		their precision (eg, 95% confidence interval). Make clear which confounders were adjusted for and why they were included<br>*Both unadjusted and adjusted estimates are presented in table 3. The confounders are described in "Statistical analysis" :age, sex, baseline NIHSS score, pre-mRS score, diabetes mellitus, hypertension in patients' history, systolic blood pressure when entering the hospital, the use of anticoagulation medication, the collaterals at CTA baseline, and the time between estimated large vessel occlusion and groin puncture.*
		(*b*) Report category boundaries when continuous variables were categorized<br>*Described in table 1 and table 2.*
		(*c*) If relevant, consider translating estimates of relative risk into absolute risk for a meaningful time period<br>*NA*
Other analyses	17	Report other analyses done—eg analyses of subgroups and interactions, and sensitivity analyses<br>*Described in "Subgroup analysis" and "supplemental 3." An interaction term was calculated to assess the interaction between occlusions location and IVT on the mRS score at 90 days.*
**Discussion**		
Key results	18	Summarise key results with reference to study objectives<br>*In this study, the use of IVT prior to EVT in patients with a posterior circulation occlusion did not lead to significant differences in clinical, technical, and safety outcomes.*
Limitations	19	Discuss limitations of the study, taking into account sources of potential bias or imprecision. Discuss both direction and magnitude of any potential bias<br>*The last paragraph of the discussion describes and summarizes the limitations of our study. Selection bias is our most important limitation.*
Interpretation	20	Give a cautious overall interpretation of results considering objectives, limitations, multiplicity of analyses, results from similar studies, and other relevant evidence<br>*In summary, our results are in line with the literature about the anterior circulation regarding IVT prior to EVT in patients with posterior circulation large vessel occlusion strokes.*
Generalisability	21	Discuss the generalisability (external validity) of the study results<br>*The generalisability is described in more detail in paragraph 3-6 in the discussion. However, no trails are performed yet on the effect of IVT prior to EVT in the posterior circulation. Randomized studies or pooling data are needed for further insights.*
**Other information**		
Funding	22	Give the source of funding and the role of the funders for the present study and, if applicable, for the original study on which the present article is based<br>*The MR CLEAN Registry (Multicenter Randomized Clinical Trial of Endovascular Treatment of Acute Ischemic Stroke) was partly funded by Stichting Toegepast Wetenschappelijk instituut voor Neuromodulatie (TWIN), Erasmus MC University Medical Center, Maastricht University Medical Center, and Amsterdam University Medical Center.*

*Give information separately for exposed and unexposed groups.

Note: An Explanation and Elaboration article discusses each checklist item and gives methodological background and published examples of transparent reporting. The STROBE checklist is best used in conjunction with this article (freely available on the Web sites of PLoS Medicine at http://www.plosmedicine.org/, Annals of Internal Medicine at

http://www.annals.org/, and Epidemiology at http://www.epidem.com/). Information on the STROBE Initiative is available at http://www.strobe-statement.org.
